# A System for Weeds and Crops Identification—Reaching over 10 FPS on Raspberry Pi with the Usage of MobileNets, DenseNet and Custom Modifications

**DOI:** 10.3390/s19173787

**Published:** 2019-08-31

**Authors:** Łukasz Chechliński, Barbara Siemiątkowska, Michał Majewski

**Affiliations:** 1 Faculty of Mechatronics, Warsaw University of Technology, 00-661 Warsaw, Poland; 2 MCMS Warka Ltd., 05-660 Warka, Poland

**Keywords:** automated weeding, mobile convolutional neural netowrks, semantic segmentation

## Abstract

Automated weeding is an important research area in agrorobotics. Weeds can be removed mechanically or with the precise usage of herbicides. Deep Learning techniques achieved state of the art results in many computer vision tasks, however their deployment on low-cost mobile computers is still challenging. The described system contains several novelties, compared both with its previous version and related work. It is a part of a project of the automatic weeding machine, developed by the Warsaw University of Technology and MCMS Warka Ltd. Obtained models reach satisfying accuracy (detecting 47–67% of weed area, misclasifing as weed 0.1–0.9% of crop area) at over 10 FPS on the Raspberry Pi 3B+ computer. It was tested for four different plant species at different growth stadiums and lighting conditions. The system performing semantic segmentation is based on Convolutional Neural Networks. Its custom architecture combines U-Net, MobileNets, DenseNet and ResNet concepts. Amount of needed manual ground truth labels was significantly decreased by the usage of the knowledge distillation process, learning final model which mimics an ensemble of complex models on a large database of unlabeled data. Further decrease of the inference time was obtained by two custom modifications: in the usage of separable convolutions in DenseNet block and in the number of channels in each layer. In the authors’ opinion, the described novelties can be easily transferred to other agrorobotics tasks.

## 1. Introduction

Weeds are considered to be one of the biggest problems in agronomy. Their adverse effect is widely known—they reduce crop yields, serve as hosts for crop diseases and also produce toxic substances. Manual removal of weeds is labour-intensive, while the use of chemicals has long-term environmental consequences [1,2,3]. It seems that automatic mechanical weeding (or alternatively precise usage of herbicides) is the solution to the problem. In recent years, there has been a significant development in the field of agricultural robotics. Automation and robotisation of agriculture allow us to reduce the number of people needed to carry out work in this sector, especially for seasonal work. Appropriate devices, differing in their degree of sophistication, cost, effectiveness and work efficiency, are described in many scientific publications.

The Greenbot [4] is an autonomous robot developed for professionals in India. It can uproot weeds in a grape field, using a high speed blade. The robot is controlled by a Raspberry Pi 2 with the usage of a camera, GPS and obstacle detecting sensor. The power source for this robot is provided through a solar panel along with a rechargeable battery, making it an environment-friendly device. Vitirover [5] is an autonomous robot designed to cut the grass and weeds between grape vines, it uses a solar panel to produce energy. A vision system is used for detecting the presence of weeds. The GPS antenna provides the coordinates for navigation of the robot. The robot OZ (developed by Naio Technologies http://www.naio-technologies.com/en/, used for tests in e.g., [6]) is an autonomous robot, provided for fully mechanical weed control. The robot is equipped with lasers and cameras. It moves autonomously and detects different types of plants. Automated weeding robots are often very costly. From economic reasons, application of robotic systems is justified only in countries with high labour costs. The system described in this paper is a part of a solution dedicated to emerging markets, prepared by MCMS Warka Ltd. in cooperation with the Faculty of Mechatronics of Warsaw University of Technology.

All agriculture robots need an effective plant localization system. Plant identification has to be both accurate and rapid. It is often done by an image analysis, but other approaches are also possible [7]. Wälchen and Mäder 2017 [8] present the first systematic review of computer vision techniques for plant identification. The traditional vision systems consist of two parts: features detection and classification. In [9,10] the scale-invariant feature transform (SIFT) is proposed, the histogram of oriented gradient (HOG) is described in [11]. Shape [12] is vital for object identification. Shape descriptors are usually combined with features like texture [13]. Many of commercially available automatic weeding solutions are based on color segmentation [14,15]. Such an approach does not enable distinguishing weeds from crops in the case when they have an equal size or they are adjacent.

The usage of convolutional networks (CNN) is a trend in the development of the next generation of weeding machines [16]. An overview of Deep Learning techniques in this field can be found in [17,18]. CNN is inspired by the organization of the animal visual cortex and is used for processing images. It allows learning spatial hierarchies of features, from low- to high-level patterns and typically consists of three types of layers: convolution, pooling, and fully connected layers. The convolution layer is an essential part of CNN. The architecture of this layer depends on the number of filters (convolution), the kernel size and the stride. Pooling layers combine the outputs of neuron clusters into a single neuron and reduce the dimensions of the data. Convolution and pooling layers perform feature extraction. A fully connected layer classifies the image based on the features. It is known that learning the features through CNN can provide better results than conventional solutions. However, these techniques rely on a massive amount of training data, and the training process is very long. In order to improve the image recognition process, new architectures of CNN have been introduced. In MobileNets [19] the operation of the convolution is divided into spatial and inter-channel parts, and the number of required calculations have been reduced several times. Introduced parameter α allows to select appropriate trade-off between network accuracy and inference time. ResNet [20] solves the vanishing gradient issue. The core idea of ResNet is introducing a so-called “identity shortcut connection” that skips one or more layers. Dense Convolutional Network (DenseNet) [21] connects each layer to every other layer in a block in a feed-forward fashion. U-Net [22] was first developed for a biomedical image segmentation tasks, but later the architecture was succesfully applied to other domains. PSPNet (Pyramid Scene Parsing Network) [23] is the champion of ImageNet Scene Parsing Challenge 2016. By using Pyramid Pooling modules, with different-region-based context aggregated, PSPNet provides a superior framework for pixel-level prediction tasks.

DeepWeeds [24] is a multiclass Australian weed species image dataset for Deep Learning. In [25] a hybrid model of AlexNet and VGGNET is proposed for crop-weed classification. DeepFruits [26] adapts Faster R-CNN to fruit detection with the usage of both colour and near-infrared images. DeepCount [27] performs a yield estimation task with a usage of Deep Learning network trained only on simulated images, without the usage of any manually labeled dataset.

The rest of the paper is organised as follows: Section 2 presents an overview of the developed weeding machine, Section 3 describes the development methodology of the vision system, Section 4 shows the experimental results while Section 5 concludes.

Automated weeding is a popular agrorobotics research topic, but to our best knowledge this is the first work describing the usage of modern mobile CNNs and a large database of unlabelled data in this domain.

## 2. Overview of the Weeding Machine

The weeding machine will be mounted on a tractor, which will result in a price lower than in the case of standalone solutions. Computational power will be provided by a cheap Raspberry Pi 3B+ microcomputer. The machine must be user-friendly and suitable for low crops growing in rows. Several modules can be connected together to enable weeding of multiple rows. Depending on the selected tool weeds can be removed mechanically, with the precise usage of herbicides or thermally (with the usage of hot water vapor). Crops growing in rows cannot be damaged, at least 90% of weeds must be removed (during regular weeding, not a single pass). The segmentation must be performed with a rate of at least 10 FPS to enable smooth device control. Camera’s field of view at the ground level is approximately 0.74 m width and 0.55 m long, so with the considered working velocity of 4 km/h, the images will strongly overlap.

**Figure 1:**
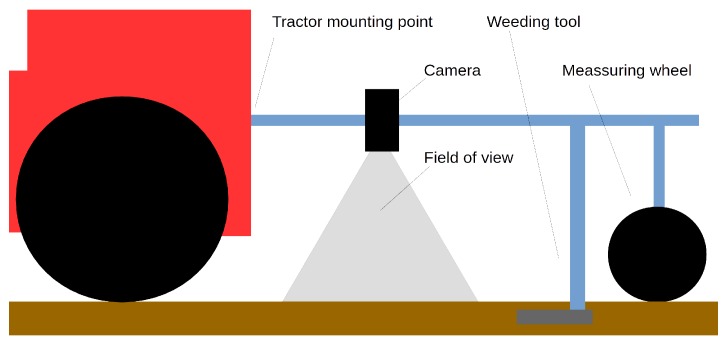
The overall scheme of a single module of the weeding machine. A camera is mounted near to the tractor, before the weeding tool. The measuring wheel position may change, as it is placed between crop rows. Note that the sketched mechanical weeding tool can be replaced with a pesticide or water vapour nozzle.

The overall scheme of the device is presented in Figure 1. The device will be mounted behind the tractor, a weeding tool will be mounted behind the camera. The machine must be dust-proof, especially in case of higher work velocities. The weeding tool is placed in a known distance from the camera field of view, so constant measurement of the machine movement is required. This measurement will be done by fusion of signals from the measuring wheel and visual odometry (from multiple modules in case of multirow weeding). The odometry error must not be larger than 1 cm on a distance of 1 m, which is the approximate distance between the camera and the weeding tool.

Camera calibration, both internal and relative to the weeding tools, will be done at the machine production stage. However, the camera to ground distance is depended on the machine-to-tractor mounting point and ground unevenness. The usage of ultrasound sonars or laser triangulation will enable to measure this parameter online.

The system of crops and weeds semantic segmentation assigns a class probability for each pixel. Control of the weeding tool is based on the segmentation output, odometry system and calibration of all subsystems. The following part of the paper will describe only the semantic segmentation, the rest of the components are beyond its scope.

## 3. Development Methodology of the Plant Segmentation System

The segmentation method has to assign probabilities of three classes (crops, weeds, and soil) to each pixel. Input resolution equals 640 × 480 pixels, and the input camera image covers a width of a single crop row. Acquisition of exactly such images was performed in the season 2018: 17 training and 7 test videos were obtained. A larger dataset (containing 92 training and 34 test videos) was acquired in the season 2017, but with a different hardware configuration (another camera model, multiple crop rows in the field of view, another input resolution), so videos were manually cropped and scaled.

**Figure 2:**
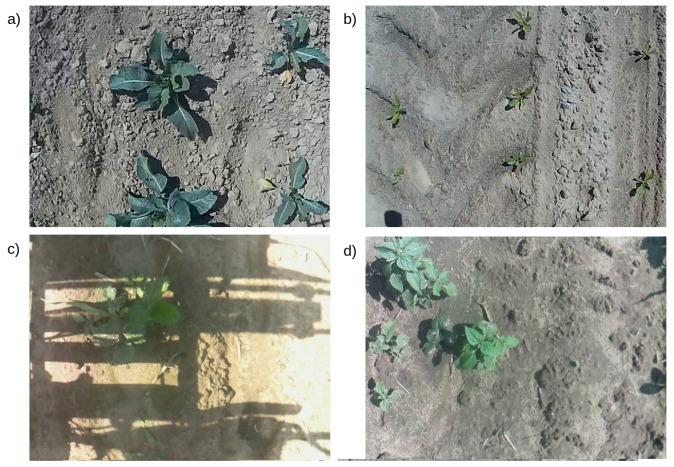
Example images from both seasons: (**a**) cauliflower 2017 (**b**) beet 2017 (**c**) strawberry 2018 (**d**) strawberry 2018. Season 2017 manually cropped and scaled. Note strong lighting and shadows in (**c**), caused by the sun orientation and casing construction.

Each video contains images from a ride along a single crop row. Four crop species (beet, cauliflower, cabbage, strawberry) at different growth stages are present. The camera is facing down the ground. Sample images from both 2017 and 2018 seasons are presented in Figure 2.

**Figure 3:**
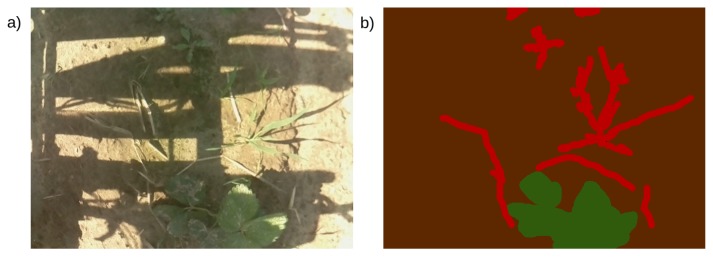
An example image (**a**) and the corresponding manual ground truth label (**b**). Crops are marked green, weeds are marked red and soil is marked brown.

Manual ground truth labels, assigning each pixel to one of three classes (crop, weed, soil), were prepared for selected training images from both seasons, with the usage of the aiding software developed within the project, named PlantLabeler. 261 labels were prepared for the season 2017 and 600 for the season 2018. A sample image with its ground truth label is presented in Figure 3. The preparation of manual labels is time-consuming, exact borders between classes are ambiguous. Moreover, the tedious character of this work causes experts’ inconsequences such as incorrect labels in less important places (plants at image borders, the soil between leaves of the same plant).

**Figure 4:**
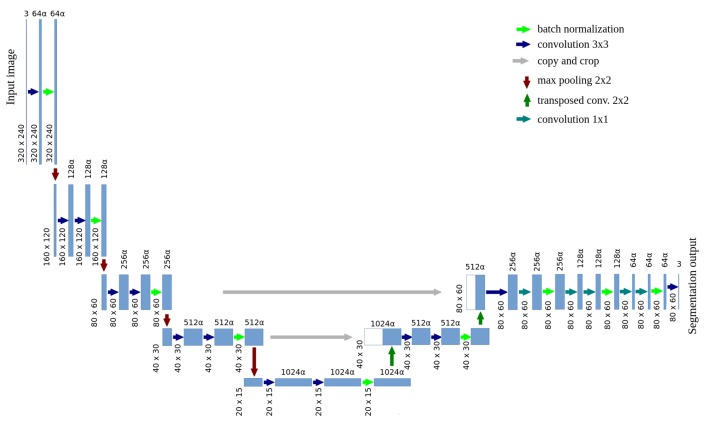
A scheme of the stationary, U-net based model, performing plants semantic segmentation.

Training of the U-net based, semantic segmentation model was conducted with the usage of manual ground truth labels. This model is presented in Figure 4 and is called stationary, in opposition to the final mobile model. Note that every instance of the segmentation model is tested for all four crop species, there are no separate models for e.g., strawberry or cabbage. The number of channels in convolutional layers depends on the parameter α, like in MobileNets [19]. The value of α=1.0 is considered as a base network structure. With lower values of α, the number of channel in each layer is lower (e.g., α=0.5 reduces number of channel from 64 to 32, from 128 to 64 etc.). Lower number of channels results in lower inference time and lower number of network parameters (which prevents from overfitting to a small training dataset, but also reduces accuracy if α is too small). The input resolution is reduced to 320 × 240 pixels (which equals about 2.3 mm/pixel on the ground level) and the output resolution was reduced to 80 × 60 pixels (about 9.2 mm/pixel). Such accuracy is satisfying in the automatic weeding task. Pixels on the borders between classes were not included during the stationary model training. Data augmentation techniques were used, including changing images brightness and saturation, as well as horizontal and vertical image flipping.

Stationary models were trained with the value of α=0.3, which enabled enough accuracy, prevented overfitting and kept training times reasonably low for complete ensembles of models. Predictions of a single model were generally correct, but they contain some noise. It was reduced with the use of ensemble learning, calculating the final probability distribution based on predictions of several independent model instances. A simple version of ensemble learning was chosen—first 50 stationary model instances were trained, and their results were averaged (usage of median was also tested, but resulted in slightly lower accuracy). A number of instances was chosen experimentally, in such a way that the output of the first half of the ensemble (25 instances) does not differ significantly from the output of the second half. Qualitative and quantitative results are presented in Section 4.

The ensemble of stationary models was used as a teacher in the knowledge distillation process. Its output class probability predictions for each pixel were a label for a student mobile neural network. It means that the student mimics the entire output distribution of the teacher, not only the index of the most probable class. Such labels were calculated for all video frames of the season 2018 (about 16,000 training and 8000 test images) and for uniformly sampled frames of the season 2017 (about 10,000 training and 10,000 test images). Please note that train images of the season 2018 strongly overlap, but the same plant is always only on one part of dataset (training or test). Overlapping images bring less new data than separated ones, so their usage can be viewed as a data augmentation technique, possibly more efficient than pure geometric and color transformations.

**Figure 5:**
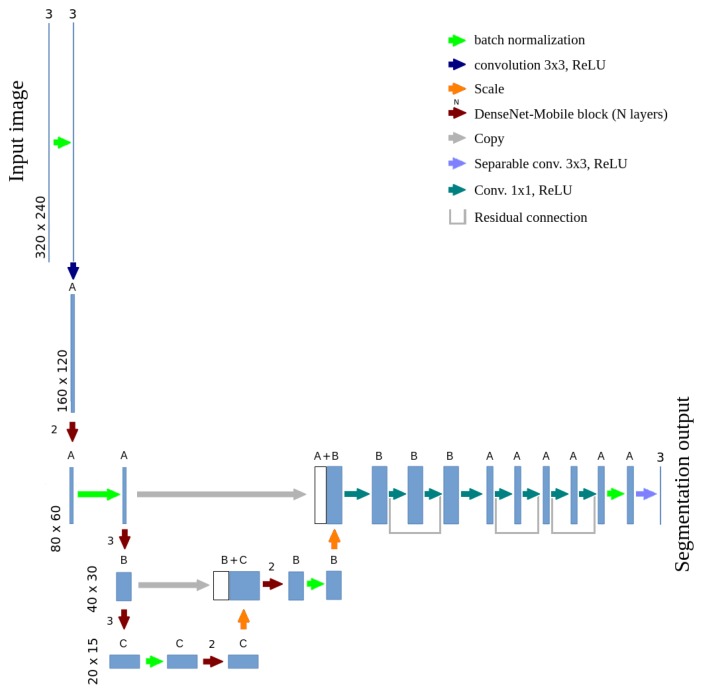
A scheme of the mobile CNN performing plant semantic segmentation, based on U-net, MobileNetsv2 and DenseNet architectures.

**Figure 6:**
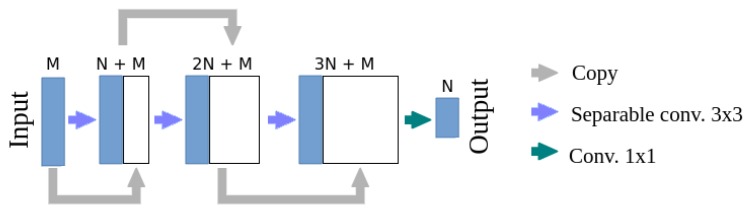
A structure of the DenseNet-Mobile block using separable convolution—naive implementation. The presented block contains three depthwise convolution layers and reduces the input resolution by a factor of two.

**Figure 7:**
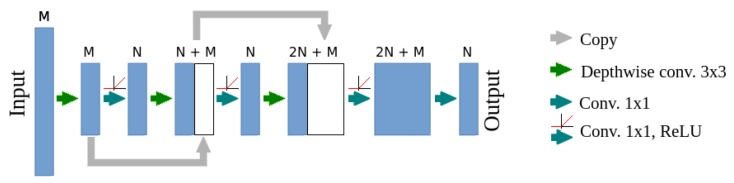
A structure of the DenseNet-Mobile block using separable convolution—custom implementation, reducing inference time. The presented block contains three depthwise convolution layers and reduces the input resolution by a factor of two.

The mobile model combines U-net, MobileNetsv2 and DenseNet architectures, its scheme is presented in Figure 5. Transposed convolutions were replaced with activation map scaling, due to the lack of the separable transposed convolution implementation in optimized software frameworks (TensorFlow 1.13 was used for the final implementation). A block of convolutional layers is the basic element of the DenseNet architecture. Its adaptation for mobile devices will be called DenseNet-Mobile. A trivial version of this adaptation is presented in Figure 6. Due to the complex structure of the DenseNet-Mobile block it can be hard to implement based on the diagram, so a short source code is presented in Appendix A.1. Simple replacement of a full convolution with a separable one results in multiple calculations of several depthwise convolutions. To avoid this, a custom solution was designed, in which the depthwise convolution is calculated once for each channel. Simultaneously, earlier reduction of resolution is possible in the case of resolution-reducing blocks, which additionally reduces the inference time. A scheme of such block is presented in Figure 7, while source code is presented in Appendix A.2. During inference of a mobile model on Raspberry Pi input was cropped to 320 × 112 pixels, which results in output resolution of 80 × 28 pixels. Such cropping allows to make prediction for each part of the crop row twice even at the machine velocity of about 4.6 km/h.

In the developed mobile architecture the number of channels in each layer depends on parameters *A*, *B* and *C* (according to Figure 5). The MobileNets architecture originally introduces a parameter α, allowing to balance between model accuracy and inference time by multiplying the number of channels in each layer by this factor. In a variant directly based on MobileNets (like in the stationary model described above) parameters *A*, *B* and *C* are defined as follows:

(1) $$\begin{array}{cc} f_{MobileNet}\left(x\right)= & min(1,\lfloor x\cdot \alpha \rfloor )\\ A= & f_{MobileNet}\left(16\right)\\ B= & f_{MobileNet}\left(32\right)\\ C= & f_{MobileNet}\left(64\right) \end{array}$$

However, tests on the Raspberry Pi 3B+ microcomputer showed that such a definition of the number of channels results in nonmonotonic inference time in function of the α parameter and in nonfunctional dependency between model accuracy and inference time. This is caused by the high efficiency of inference for layers which number of channels is a power of two or a sum of few powers of two. For example, reducing the number of channels from A=16, B=32, C=64 to A=15, B=31, C=62 results in increase of the inference time. There are few α values that allow keeping an effective number of channels in each layer. To overcome this problem, a custom formula was introduced, which defines the number of channels in each layer as a multiple of 8. The value of 8 was chosen experimentally, as the smallest one allowing for monotonic inference time in function of the parameter α. In the custom version, the number of channels in each layer is defined as:

(2) $$\begin{array}{cc} f_{mod8}\left(x\right)= & min(8,8\cdot \left\lfloor \frac{x}{8}\cdot \alpha \right\rceil )\\ A= & f_{mod8}\left(16\right)\\ B= & f_{mod8}\left(32\right)\\ C= & f_{mod8}\left(64\right) \end{array}$$

Please note that the custom modification of α parameter was applied only to the mobile architecture, as inference time of the stationary architecture is not crucial and was not optimized.

Segmentation error $e_{dice}$ is defined as 100% minus the average (for all *C* classes and *N* test images) value of the Dice coefficient (modification of IoU segmentation score, which is more suitable for direct optimization, often used in medical image segmentation evaluation [28]), which equals to the quotient of the doubled area of the common part of the label *E* and the prediction *P* over the sum of them:

(3) $$e_{dice}=100\%-\frac{1}{N\cdot C}\sum_{i=1}^{N}\sum_{j=1}^{C}\frac{2|E\cap P|}{\left|E|+|P\right|}$$

Segmentation error $e_{dice}$ measures overall accuracy of the model, but some detailed measures are more intuitive for automated weeding task. The area of correctly detected weeds is described by weed detection recall $r_{weed}$:

(4) $$r_{weed}=\frac{|E_{weed}\cap P_{weed}|}{|E_{weed}|}$$

On the other hand, weed detection precision $p_{weed}$ is useful for pesticides waste monitoring:

(5) $$p_{weed}=\frac{|E_{weed}\cap P_{weed}|}{|P_{weed}|}$$

Finally, crop yield will be significantly reduced if crop is misclassified as weed, which is measured with $m_{crop\;as\;weed}$:

(6) $$m_{crop\;as\;weed}=\frac{|E_{crop}\cap P_{weed}|}{|E_{crop}|}$$

Some other measures, e.g., crop detection precision and recall can be also useful, but their influence to yield may depend on the weeding tool control algorithm. Please note that both partial detection of weed or partial misclassification of crop as weed will influence the yield, so further evaluation is required while the machine is developed.

## 4. Experimental Results

**Figure 8:**
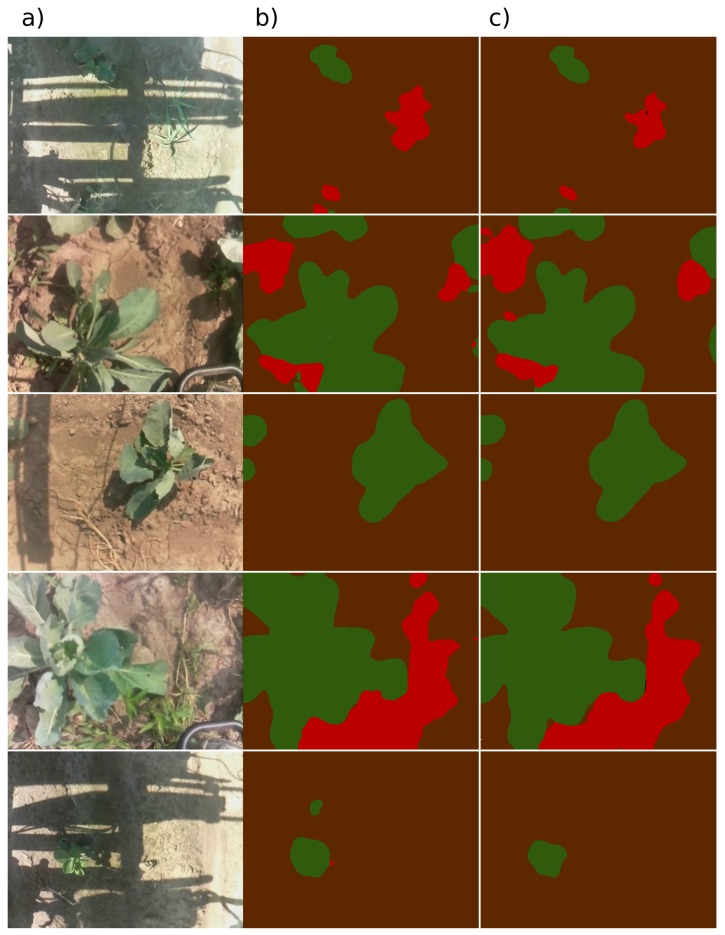
Example predictions of the stationary model: (**a**) input images (**b**) segmentation results for a single model instance (**c**) segmentation results for an ensemble of 50 model instances (output averaging). Pixels with the highest probability for crops are marked green, weeds are marked red and soil is marked brown.

Sample predictions of a single stationary model and the models’ ensemble for different input images are presented in Figure 8. Quantitative results, calculated for a validation set of additional 100 manually-labelled images, are presented in Table 1.

Experiments were performed on Raspberry Pi 3B+ with the use of TensorFlow 1.13 Deep Learning framework. A version of the framework binaries dedicated for Raspberry Pi was provided by the framework maintainer, so no custom build or JIT compilation was performed. The reported results were obtained for 32-bit floating point precision of all tensors. Usage of 16-bit floating point precision or weight quantization resulted in higher processing times, because it does not use highly optimized tensor-processing libraries. No other “tricks” (e.g. constant folding, network pruning) were tested.

**Figure 9:**
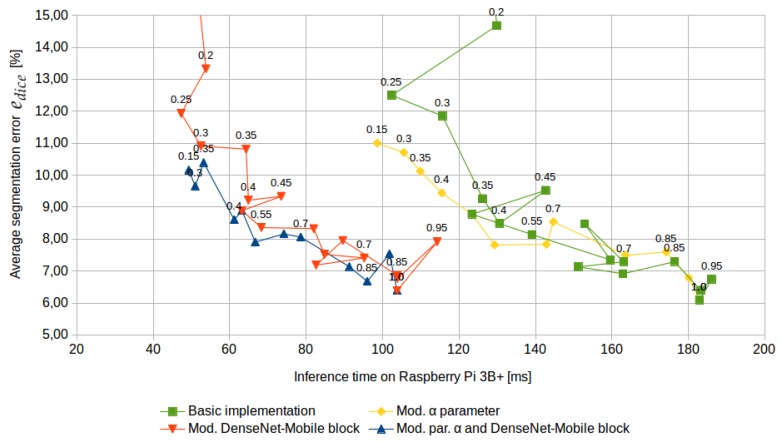
Segmentation error $e_{dice}$ and inference time for different values of α (α value captions were placed above selected points). The measurement points were connected with lines to show the order of α changes. Without the custom definition of the α parameter, the dependency between segmentation error and inference time is nonfunctional. Without the custom version of the DenseNet-Mobile block inference time is significantly higher.

**Figure 10:**
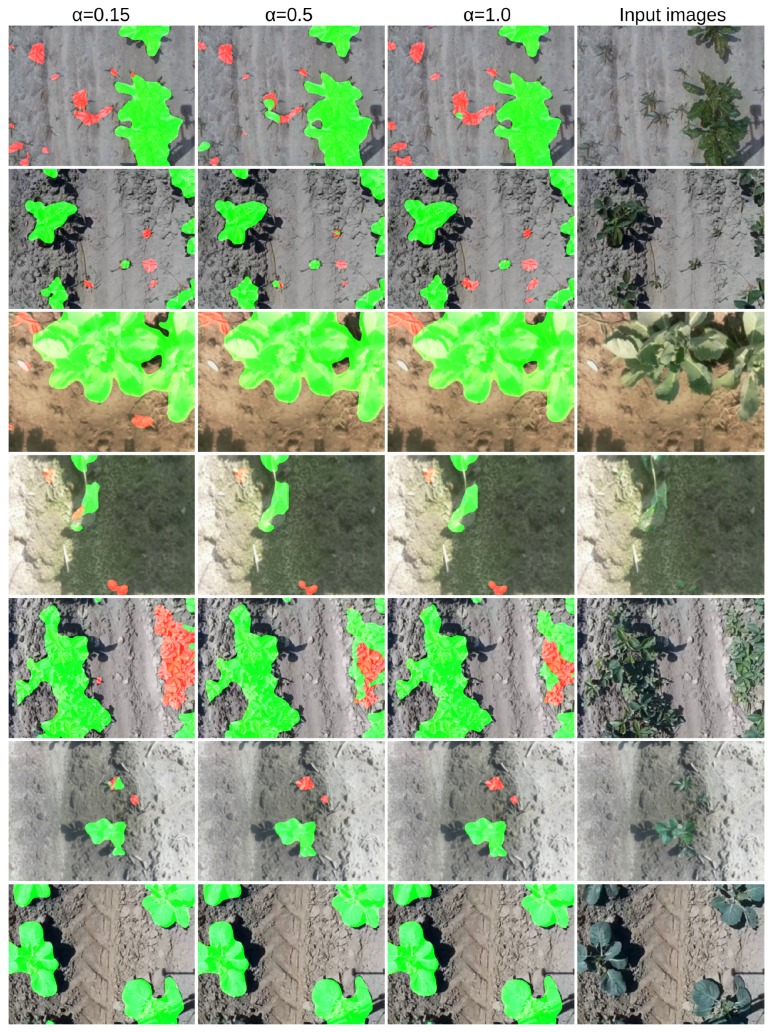
Example prediction results of the mobile model with a custom structure of the DenseNet-Mobile block and a custom definition of the α parameter. The columns show images with highlighted prediction results for different values of the α parameter, the input image is presented in the right-most column. Crops are highlighted green and weeds are highlighted red.

Inference times and segmentation errors for different α values and four architecture versions are presented in Figure 9. Sample prediction results are presented in Figure 10. The custom modification of the α parameter definition resulted in functional dependency between inference time and segmentation accuracy, while the custom structure of a DenseNet-Mobile block resulted in a significant reduction of inference time.

Note that segmentation error was also measured for the best configuration of mobile architecture (with the custom parameter α and DenseNet-Mobile block), but with direct training on manual labels. In such case for α=1.0 the average segmentation error ranges between 18.3–19.4% (training was repeated twice), which is out of range of Figure 9. The use of knowledge distillation technique reduces error to 6.4% for the same network structure.

The segmentation error in Figure 9 is based on the difference between a mobile model and the ensemble of stationary models, due to difficulties in obtaining a large database of high-quality manual labels. Parameters $e_{dice}$, $p_{weed}$, $r_{weed}$ and $m_{crop\;as\;weed}$ were also calculated on the validation dataset of 100 manually-labelled images. The results are presented in Table 2. Note that the validation dataset is much smaller than the test set (labelled by an ensemble of stationary models), so the results may have higher uncertainty.

## 5. Conclusions

The described novelties allowed to reach satisfying accuracy at low inference time which enables efficient control of the weeding machine (50–100 ms per frame on Raspberry Pi 3B+). Intensive tests will be required once the final mechanical structure of the device is developed (which is planned in 2020), including different plant species, vegetation levels, and weather conditions. In case of difficulties in obtaining satisfying accuracy in the final conditions the following remedies can be used:

- Training of different models for different plant species and growth stadiums.
- Change of the α parameter value.
- Change of the number of final residual layers.
- Reduction of the number of batch normalization operations.

The recall of weed detection is a parameter that should be optimized in further work. Note that weeding should be repeated periodically, so missed weed can be detected during the next pass.

These solutions were proposed according to the risk management rules for research projects. However, in the authors’ opinion, the probability of having to use them is low.

## Figures and Tables

**Table 1:** Quantitative results of stationary network architecture.

Configuration	$e_{\mathrm{dice}}$ [%]	$r_{\mathrm{weed}}$ [%]	$p_{\mathrm{weed}}$ [%]	$m_{\mathrm{crop}\;\mathrm{as}\;\mathrm{weed}}$ [%]
Single model—average results	3.55	53.8	60.2	1.0
Ensemble of 50 instances—mean	1.68	72.9	66.3	0.2
Ensemble of 50 instances—media	1.71	74.0	65.0	0.2

**Table 2:** Quantitative results of the mobile network architecture, with modified parameter α and DenseNet-Mobile block.

α	$e_{\mathrm{dice}}$ [%]	$r_{\mathrm{weed}}$ [%]	$p_{\mathrm{weed}}$ [%]	$m_{\mathrm{crop}\;\mathrm{as}\;\mathrm{weed}}$ [%]
0.15	2.66	67.2	50.5	0.9
0.3	2.49	70.7	54.7	0.2
0.35	2.28	79.3	47.4	0.2
0.4	2.32	76.8	57.1	0.3
0.5	2.07	77.9	55.0	0.2
0.6	2.04	76.9	60.3	0.4
0.65	1.92	76.8	63.1	0.5
0.7	2.20	75.1	52.1	0.1
0.75	2.09	70.6	66.8	0.6
0.85	2.19	68.3	53.8	0.3
0.9	2.45	77.2	48.9	0.1
1.0	2.05	74.3	55.9	0.1

## Appendix A

### Appendix A.1.

TensorFlow 1.13 source code for DenseNet-Mobile block—naive implementation

def densenetmobile_naive(input, num_filters, layers, stride=1,
                          activation=tf.nn.relu):
    prev_cat = input
    for i in range(layers + 1):
      conv = tf.layers.separable_conv2d(prev_cat, filters=num_filters,
           kernel_size=3, strides=1, activation=activation,
           padding=’same’,
           depthwise_initializer=tf.initializers.he_normal(),
           pointwise_initializer=tf.initializers.he_normal())
      prev_cat = tf.concat((prev_cat, conv), −1)
    conv_bottleneck = tf.layers.conv2d(prev_cat, filters=num_filters,
      kernel_size=1, strides=stride, activation=None, padding=‘same’,
      kernel_initializer=tf.initializers.he_normal())
    return conv_bottleneck

### Appendix A.2.

TensorFlow 1.13 source code for DenseNet-Mobile block—modified implementation

def densenetmobile_modified(input, num_filters, layers, stride=1,
				activation=tf.nn.relu, name=’layer_name’):
	strides = [1, stride, stride, 1]
	filters_input = tf.get_variable(name + ’depthinput/kernel’,
				[3, 3, input.shape[−1], 1],
				tf.float32, tf.initializers.he_normal())
	conv_depth_input = tf.nn.depthwise_conv2d(input, filter=filters_input,
					strides=strides, padding=’SAME’,
					name=name+’depthinput’)
	concated_depth = conv_depth_input
	for i in range(layers):
		letter = chr(ord(’A’) + i)
		conv_1x1 = tf.layers.conv2d(concated_depth, filters=num_filters,
				kernel_size=1, strides=1, activation=activation,
				padding=’same’, name=name + ’_1x1_’ + letter,
				kernel_initializer=tf.initializers.he_normal())
		filters = tf.get_variable(name + ’_depth_’ + letter + ’/kernel’,
				[3, 3, num_filters, 1], tf.float32,
				tf.initializers.he_normal())
		conv_depth = tf.nn.depthwise_conv2d(conv_1x1, filter=filters,
				strides=[1, 1, 1, 1], padding=’SAME’,
				name=name + letter)
		concated_depth = tf.concat((concated_depth, conv_depth), −1)
	conv_block = tf.layers.conv2d(concated_depth, filters=layers * \
				num_filters, kernel_size=1, strides=1,
				activation=activation, padding=’same’,
				name=name + ’_block’,
				kernel_initializer=tf.initializers.he_normal())
	conv_bottleneck = tf.layers.conv2d(conv_block, filters=num_filters,
				kernel_size=1, strides=1, activation=None,
				padding=’same’, name=name + ’_bottleneck’,
				kernel_initializer=tf.initializers.he_normal())
	return conv_bottleneck
